# Missing the Dark: Health Effects of Light Pollution

**DOI:** 10.1289/ehp.117-a20

**Published:** 2009-01

**Authors:** Ron Chepesiuk

In 1879, Thomas Edison’s incandescent light bulbs first illuminated a New York street, and the modern era of electric lighting began. Since then, the world has become awash in electric light. Powerful lamps light up streets, yards, parking lots, and billboards. Sports facilities blaze with light that is visible for tens of miles. Business and office building windows glow throughout the night. According to the Tucson, Arizona–based International Dark-Sky Association (IDA), the sky glow of Los Angeles is visible from an airplane 200 miles away. In most of the world’s large urban centers, stargazing is something that happens at a planetarium. Indeed, when a 1994 earthquake knocked out the power in Los Angeles, many anxious residents called local emergency centers to report seeing a strange “giant, silvery cloud” in the dark sky. What they were really seeing—for the first time—was the Milky Way, long obliterated by the urban sky glow.

None of this is to say that electric lights are inherently bad. Artificial light has benefited society by, for instance, extending the length of the productive day, offering more time not just for working but also for recreational activities that require light. But when artificial outdoor lighting becomes inefficient, annoying, and unnecessary, it is known as light pollution. Many environmentalists, naturalists, and medical researchers consider light pollution to be one of the fastest growing and most pervasive forms of environmental pollution. And a growing body of scientific research suggests that light pollution can have lasting adverse effects on both human and wildlife health.

When does nuisance light become a health hazard? Richard Stevens, a professor and cancer epidemiologist at the University of Connecticut Health Center in Farmington, Connecticut, says light photons must hit the retina for biologic effects to occur. “However, in an environment where there is much artificial light at night—such as Manhattan or Las Vegas—there is much more opportunity for exposure of the retina to photons that might disrupt circadian rhythm,” he says. “So I think it is not only ‘night owls’ who get those photons. Almost all of us awaken during the night for periods of time, and unless we have blackout shades there is some electric lighting coming in our windows. It is not clear how much is too much; that is an important part of the research now.”

According to “The First World Atlas of the Artificial Night Sky Brightness,” a report on global light pollution published in volume 328, issue 3 (2001) of the *Monthly Notices of the Royal Astronomical Society*, two-thirds of the U.S. population and more than one-half of the European population have already lost the ability to see the Milky Way with the naked eye. Moreover, 63% of the world population and 99% of the population of the European Union and the United States (excluding Alaska and Hawaii) live in areas where the night sky is brighter than the threshold for light-polluted status set by the International Astronomical Union—that is, the artificial sky brightness is greater than 10% of the natural sky brightness above 45° of elevation.

Light pollution comes in many forms, including sky glow, light trespass, glare, and over illumination. Sky glow is the bright halo that appears over urban areas at night, a product of light being scattered by water droplets or particles in the air. Light trespass occurs when unwanted artificial light from, for instance, a floodlight or streetlight spills onto an adjacent property, lighting an area that would otherwise be dark. Glare is created by light that shines horizontally. Overillumination refers to the use of artificial light well beyond what is required for a specific activity, such as keeping the lights on all night in an empty office building.

## Distracted by the Light

The ecologic effects of artificial light have been well documented. Light pollution has been shown to affect both flora and fauna. For instance, prolonged exposure to artificial light prevents many trees from adjusting to seasonal variations, according to Winslow Briggs’s chapter on plant responses in the 2006 book *Ecological Consequences of Artificial Night Lighting*. This, in turn, has implications for the wildlife that depend on trees for their natural habitat. Research on insects, turtles, birds, fish, reptiles, and other wildlife species shows that light pollution can alter behaviors, foraging areas, and breeding cycles, and not just in urban centers but in rural areas as well.

Sea turtles provide one dramatic example of how artificial light on beaches can disrupt behavior. Many species of sea turtles lay their eggs on beaches, with females returning for decades to the beaches where they were born to nest. When these beaches are brightly lit at night, females may be discouraged from nesting in them; they can also be disoriented by lights and wander onto nearby roadways, where they risk being struck by vehicles.

Moreover, sea turtle hatchlings normally navigate toward the sea by orienting away from the elevated, dark silhouette of the landward horizon, according to a study published by Michael Salmon of Florida Atlantic University and colleagues in volume 122, number 1–2 (1992) of *Behaviour*. When there are artificial bright lights on the beach, newly hatched turtles become disoriented and navigate toward the artificial light source, never finding the sea.

Jean Higgins, an environmental specialist with the Florida Wildlife Conservation Commission Imperiled Species Management Section, says disorientation also contributes to dehydration and exhaustion in hatchlings. “It’s hard to say if the ones that have made it into the water aren’t more susceptible to predation at this later point,” she says.

Bright electric lights can also disrupt the behavior of birds. About 200 species of birds fly their migration patterns at night over North America, and especially during inclement weather with low cloud cover, they routinely are confused during passage by brightly lit buildings, communication towers, and other structures. “Light attracts birds and disorients them,” explains Michael Mesure, executive director of the Toronto-based Fatal Light Awareness Program (FLAP), which works to safeguard migratory birds in the urban environment. “It is a serious situation because many species that collide frequently are known to be in long-term decline and some are already designated officially as threatened.”

Each year in New York City alone, about 10,000 migratory birds are injured or killed crashing into skyscrapers and high-rise buildings, says Glenn Phillips, executive director of the New York City Audubon Society. The estimates as to the number of birds dying from collisions across North America annually range from 98 million to close to a billion. The U.S. Fish and Wildlife Service estimates 5–50 million birds die each year from collisions with communication towers.

Turtles and birds are not the only wildlife affected by artificial nighttime lighting. Frogs have been found to inhibit their mating calls when they are exposed to excessive light at night, reducing their reproductive capacity. The feeding behavior of bats also is altered by artificial light. Researchers have blamed light pollution for declines in populations of North American moths, according to *Ecological Consequences of Artificial Night Lighting*. Almost all small rodents and carnivores, 80% of marsupials, and 20% of primates are nocturnal. “We are just now understanding the nocturnality of many creatures,” says Chad Moore, Night Sky Program manager with the National Park Service. “Not protecting the night will destroy the habitat of many animals.”

## Resetting the Circadian Clock

The health effects of light pollution have not been as well defined for humans as for wildlife, although a compelling amount of epidemiologic evidence points to a consistent association between exposure to indoor artificial nighttime light and health problems such as breast cancer, says George Brainard, a professor of neurology at Jefferson Medical College, Thomas Jefferson University in Philadelphia. “That association does not prove that artificial light causes the problem. On the other hand, controlled laboratory studies do show that exposure to light during the night can disrupt circadian and neuroendocrine physiology, thereby accelerating tumor growth.”

The 24-hour day/night cycle, known as the circadian clock, affects physiologic processes in almost all organisms. These processes include brain wave patterns, hormone production, cell regulation, and other biologic activities. Disruption of the circadian clock is linked to several medical disorders in humans, including depression, insomnia, cardiovascular disease, and cancer, says Paolo Sassone-Corsi, chairman of the Pharmacology Department at the University of California, Irvine, who has done extensive research on the circadian clock. “Studies show that the circadian cycle controls from ten to fifteen percent of our genes,” he explains. “So the disruption of the circadian cycle can cause a lot of health problems.”

On 14–15 September 2006 the National Institute of Environmental Health Sciences (NIEHS) sponsored a meeting that focused on how best to conduct research on possible connections between artificial lighting and human health. A report of that meeting in the September 2007 issue of *EHP* stated, “One of the defining characteristics of life in the modern world is the altered patterns of light and dark in the built environment made possible by use of electric power.” The meeting report authors noted it may not be entirely coincidental that dramatic increases in the risk of breast and prostate cancers, obesity, and early-onset diabetes have mirrored the dramatic changes in the amount and pattern of artificial light generated during the night and day in modern societies over recent decades. “The science underlying these hypotheses has a solid base,” they wrote, “and is currently moving forward rapidly.”

The connection between artificial light and sleep disorders is a fairly intuitive one. Difficulties with adjusting the circadian clock can lead to a number of sleep disorders, including shift-work sleep disorder, which affects people who rotate shifts or work at night, and delayed sleep–phase syndrome, in which people tend to fall asleep very late at night and have difficulty waking up in time for work, school, or social engagements.

The sleep pattern that was the norm before the invention of electric lights is no longer the norm in countries where artificial light extends the day. In the 2005 book *At Day’s Close: Night in Times Past*, historian Roger Ekirch of Virginia Polytechnic Institute described how before the Industrial Age people slept in two 4-hour shifts (“first sleep” and “second sleep”) separated by a late-night period of quiet wakefulness.

Thomas A. Wehr, a psychiatrist at the National Institute of Mental Health, has studied whether humans would revert back to the two-shift sleep pattern if they were not exposed to the longer photoperiod afforded by artificial lighting. In the June 1992 *Journal of Sleep Research*, Wehr reported his findings on eight healthy men, whose light/dark schedule was shifted from their customary 16 hours of light and 8 hours of dark to a schedule in which they were exposed to natural and electric light for 10 hours, then darkness for 14 hours to simulate natural durations of day and night in winter. The subjects did indeed revert to the two-shift pattern, sleeping in two sessions of about 4 hours each separated by 1–3 hours of quiet wakefulness.

## Beyond Sleep Disorders

Alteration of the circadian clock can branch into other effects besides sleep disorders. A team of Vanderbilt University researchers considered the possibility that constant artificial light exposure in neonatal intensive care units could impair the developing circadian rhythm of premature babies. In a study published in the August 2006 issue of *Pediatric Research*, they exposed newborn mice (comparable in development to 13-week-old human fetuses) to constant artificial light for several weeks. The exposed mice were were unable to maintain a coherent circadian cycle at age 3 weeks (comparable to a full-term human neonate). Mice exposed for an additional 4 weeks were unable to establish a regular activity cycle. The researchers concluded that excessive artificial light exposure early in life might contribute to an increased risk of depression and other mood disorders in humans. Lead researcher Douglas McMahon notes, “All this is speculative at this time, but certainly the data would indicate that human infants benefit from the synchronizing effect of a normal light/dark cycle.”

Since 1995, studies in such journals as *Epidemiology, Cancer Causes and Control*, the *Journal of the National Cancer Institute*, and *Aviation Space Environmental Medicine*, among others, have examined female employees working a rotating night shift and found that an elevated breast cancer risk is associated with occupational exposure to artificial light at night. Mariana Figueiro, program director at the Lighting Research Center of Rensselaer Polytechnic Institute in Troy, New York, notes that permanent shift workers may be less likely to be disrupted by night work because their circadian rhythm can readjust to the night work as long as light/dark patterns are controlled.

In a study published in the 17 October 2001 *Journal of the National Cancer Institute*, Harvard University epidemiologist Eva S. Schernhammer and colleagues from Brigham and Women’s Hospital in Boston used data from the 1988 Nurses’ Health Study (NHS), which surveyed 121,701 registered female nurses on a range of health issues. Schernhammer and her colleagues found an association between breast cancer and shift work that was restricted to women who had worked 30 or more years on rotating night shifts (0.5% of the study population).

In another study of the NHS cohort, Schernhammer and colleagues also found elevated breast cancer risk associated with rotating night shift work. Discussing this finding in the January 2006 issue of *Epidemiology*, they wrote that shift work was associated with only a modest increased breast cancer risk among the women studied. The researchers further wrote, however, that their study’s findings “in combination with the results of earlier work, reduce the likelihood that this association is due solely to chance.”

Schernhammer and her colleagues have also used their NHS cohort to investigate the connection between artificial light, night work, and colorectal cancer. In the 4 June 2003 issue of the *Journal of the National Cancer Institute*, they reported that nurses who worked night shifts at least 3 times a month for 15 years or more had a 35% increased risk of colorectal cancer. This is the first significant evidence so far linking night work and colorectal cancer, so it’s too early to draw conclusions about a causal association. “There is even less evidence about colorectal cancer and the larger subject of light pollution,” explains Stevens. “That does not mean there is no effect, but rather, there is not enough evidence to render a verdict at this time.”

The research on the shift work/cancer relationship is not conclusive, but it was enough for the International Agency for Research on Cancer (IARC) to classify shift work as a probable human carcinogen in 2007. “The IARC didn’t definitely call night shift work a carcinogen,” Brainard says. “It’s still too soon to go there, but there is enough evidence to raise the flag. That’s why more research is still needed.”

## The Role of Melatonin

Brainard and a growing number of researchers believe that melatonin may be the key to understanding the shift work/breast cancer risk association. Melatonin, a hormone produced by the pineal gland, is secreted at night and is known for helping to regulate the body’s biologic clock. Melatonin triggers a host of biologic activities, possibly including a nocturnal reduction in the body’s production of estrogen. The body produces melatonin at night, and melatonin levels drop precipitously in the presence of artificial or natural light. Numerous studies suggest that decreasing nocturnal melatonin production levels increases an individual’s risk of developing cancer. [For more information on melatonin, see “Benefits of Sunlight: A Bright Spot for Human Health,” *EHP* 116:A160–A167 (2008).]

One groundbreaking study published in the 1 December 2005 issue of *Cancer Research* implicated melatonin deficiency in what the report authors called a rational biologic explanation for the increased breast cancer risk in female night shift workers. The study involved female volunteers whose blood was collected under three different conditions: during daylight hours, during the night after 2 hours of complete darkness, and during the night after exposure to 90 minutes of artificial light. The blood was injected into human breast tumors that were transplanted into rats. The tumors infused with melatonin-deficient blood collected after exposure to light during the night were found to grow at the same speed as those infused with daytime blood. The blood collected after exposure to darkness slowed tumor growth.

“We now know that light suppresses melatonin, but we are not saying it is the only risk factor,” says first author David Blask, a research scientist at the Bassett Healthcare Research Institute in Cooperstown, New York. “But light is a risk factor that may explain [previously unexplainable phenomena]. So we need to seriously consider it.”

The National Cancer Institute estimates that 1 in 8 women will be diagnosed with breast cancer at some time during her life. We can attribute only about half of all breast cancer cases to known risk factors, says Brainard. Meanwhile, he says, the breast cancer rate keeps climbing—incidence increased by more than 40% between 1973 and 1998, according to the Breast Cancer Fund—and “we need to understand what’s going on as soon as possible.”

## Linking Light Pollution to Human Health

The evidence that indoor artificial light at night influences human health is fairly strong, but how does this relate to light pollution? The work in this area has just begun, but two studies in Israel have yielded some intriguing findings. Stevens was part of a study team that used satellite photos to gauge the level of nighttime artificial light in 147 communities in Israel, then overlaid the photos with a map detailing the distribution of breast cancer cases. The results showed a statistically significant correlation between outdoor artificial light at night and breast cancer, even when controlling for population density, affluence, and air pollution. Women living in neighborhoods where it was bright enough to read a book outside at midnight had a 73% higher risk of developing breast cancer than those residing in areas with the least outdoor artificial lighting. However, lung cancer risk was not affected. The findings appeared in the January 2008 issue of *Chronobiology International*.

“It may turn out that artificial light exposure at night increases risk, but not entirely by the melatonin mechanism, so we need to do more studies of ‘clock’ genes—nine have so far been identified—and light exposure in rodent models and humans,” Stevens says. Clock genes carry the genetic instructions to produce protein products that control circadian rhythm. Research needs to be done not just on the light pollution–cancer connection but also on several other diseases that may be influenced by light and dark.

Travis Longcore, co-editor of *Ecological Consequences of Artificial Night Lighting* and a research associate professor at the University of Southern California Center for Sustainable Cities, suggests two ways outdoor light pollution may contribute to artificial light–associated health effects in humans. “From a human health perspective, it seems that we are concerned with whatever increases artificial light exposure indoors at night,” he says. “The effect of outdoor lighting on indoor exposure could be either direct or indirect. In the direct impact scenario, the artificial light from outside reaches people inside at night at levels that affect production of hormones. In an indirect impact it would disturb people inside, who then turn on lights and expose themselves to more light.”

“The public needs to know about the factors causing [light pollution], but research is not going at the pace it should,” Blask says. Susan Golden, distinguished professor at the Center for Research on Biological Clocks of Texas A&M University in College Station, Texas, agrees. She says, “Light pollution is still way down the list of important environmental issues needing study. That’s why it’s so hard to get funds to research the issue.”

“The policy implications of unnecessary light at night are enormous,” says Stevens in reference to the health and energy ramifications [for more on the energy impact of light pollution, see “Switch On the Night: Policies for Smarter Lighting,” p. A28 this issue]. “It is fully as important an issue as global warming.” Moreover, he says, artificial light is a ubiquitous environmental agent. “Almost everyone in modern society uses electric light to reduce the natural daily dark period by extending light into the evening or before sunrise in the morning,” he says. “On that basis, we are all exposed to electric light at night, whereas before electricity, and still in much of the developing world, people get twelve hours of dark whether they are asleep or not.”

Sources believe that the meeting at the NIEHS in September 2006 was a promising beginning for moving forward on the light pollution issue. “Ten years ago, scientists thought something was there, but couldn’t put a finger on it,” says Leslie Reinlib, a program director at the NIEHS who helped organize the meeting. “Now we are really just at the tip of the iceberg, but we do have something that’s scientific and can be measured.”

The 23 participants at the NIEHS-sponsored meeting identified a research agenda for further study that included the functioning of the circadian clock, epidemiologic studies to define the artificial light exposure/disease relationship, the role of melatonin in artificial light–induced disease, and development of interventions and treatments to reduce the impact of light pollution on disease. “It was a very significant meeting,” Brainard says. “It’s the first time the National Institutes of Health sponsored a broad multidisciplinary look at the light-environmental question with the intent of moving to the next step.”

## Figures and Tables

**Figure:**
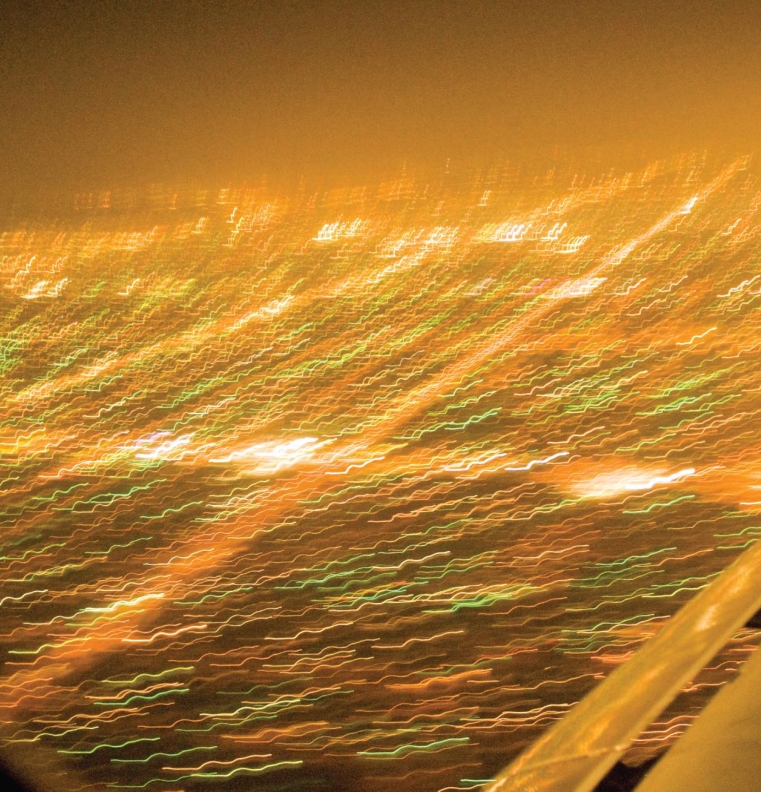


**Figure:**
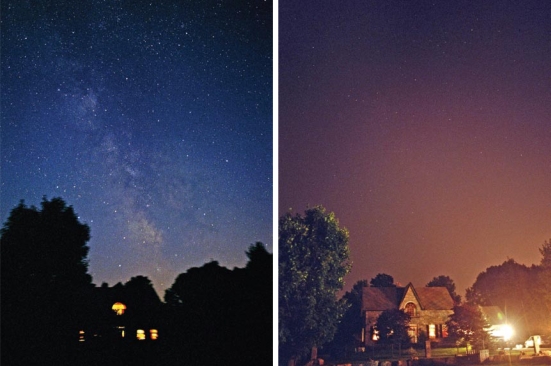
Glare, overillumination, and sky glow (which makes the sky over a city look orange, yellow, or pink) are all forms of light pollution. These photos were taken in Goodwood, Ontario, a small town about 45 minutes northeast of Toronto during and the night after the regionwide 14 August 2003 blackout. The lights inside the house in the blackout picture were created by candles and flashlights.

**Figure:**
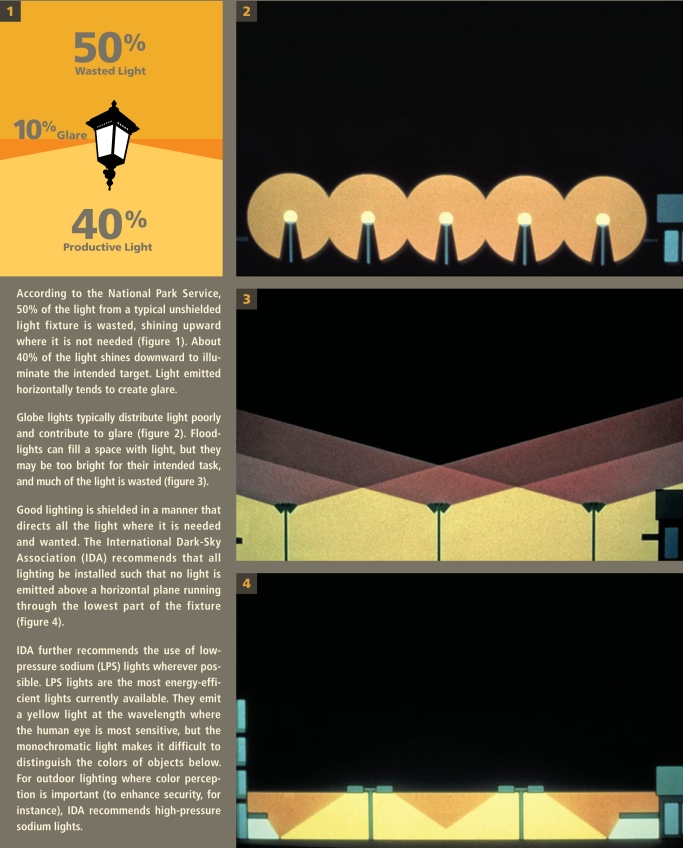
How Outdoor Lighting Translates into Light Pollution

**Figure:**
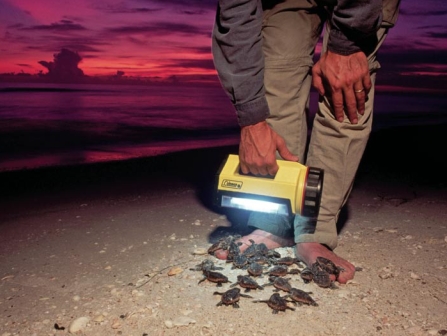
Turtle hatchlings instinctively orient away from the dark silhouette of the nighttime shore. Here hatchlings have been temporarily distracted by a bright lamp. Hatchlings and mother turtles distracted by shorefront lights can wander onto nearby roadways.

**Figure:**
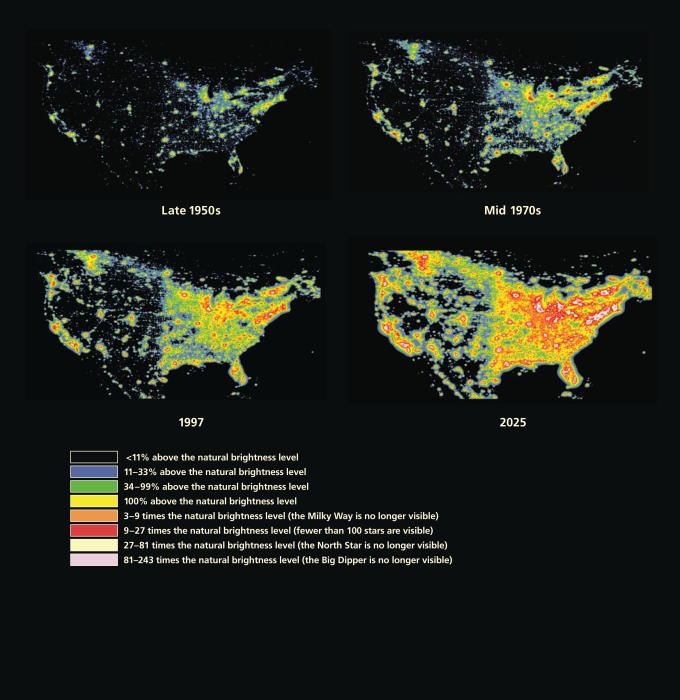
Increase in Artificial Night Sky Brightness in North America

**Figure:**
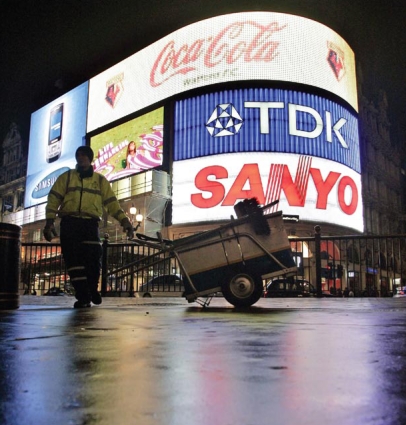
The International Agency for Research on Cancer has classified shift work as a probable human carcinogen. A study in the December 2008 issue of Sleep found that use of light exposure therapy, sunglasses, and a strict sleep schedule may help night-shift workers achieve a better-balanced circadian rhythm.

